# ALK阳性非小细胞肺癌的诊断

**DOI:** 10.3779/j.issn.1009-3419.2015.02.02

**Published:** 2015-02-20

**Authors:** 勤 冯, 欣 杨, 冬梅 林

**Affiliations:** 100142 北京，北京大学肿瘤医院病理科暨北京市肿瘤防 治研究所恶性肿瘤发病机制及转教育部重点实验室 Department of Pathology, Key Laboratory of Carcinogenesis and Translational Research (Ministry of Education), Peking University Cancer Hospital and Institute, Beijing 100142, China

**Keywords:** 肺肿瘤, 间变性淋巴瘤激酶, 荧光原位杂交, 免疫组织化学, 聚合酶链反应, Lung neoplasms, Anaplastic lymphoma kinase, Fluorescence *in situ* hybridization, Immunohistochemistry, Polymerase chain reaction

## Abstract

间变性淋巴瘤激酶（anaplastic lymphoma kinase, *ALK*）融合基因是非小细胞肺癌（non-small cell lung cancer, NSCLC）的驱动基因，存在此基因表达的ALK阳性NSCLC已被认为是NSCLC的一种分子亚型，具有较独特的临床病理特征和预后，更为重要的是，ALK抑制剂对于此类晚期患者具有明显的疗效。因此，如何诊断此类患者便成为了病理诊断工作中相当重要的部分。目前ALK阳性NSCLC的诊断方法较为多样且各有优缺点，如何挑选最佳方法用于肺癌样本的常规诊断成为临床病理所关注的重点。国内外已有较多权威指南或共识推荐了诊断方法及其流程或相关标准。基于此，本文对ALK阳性NSCLC的诊断方法及特殊检测样本类型做一综述。

## Abstract (en)

The anaplastic lymphoma kinase (*ALK*) gene rearrangement is a driver gene of non-small cell lung cancer (NSCLC). Positive expression of *ALK* gene rearrangement has been considered a molecular subtype of NSCLC, that there are special pathological characteristics and clinical prognosis. Furthermore, the tyrosine kinase inhibitor has demonstrated high effective in treating ALK positive NSCLC patients. Thus making molecular diagnostic testing for ALK expression is an important step in the process of pathological diagnosis. Currently, many detecting ALK expression assays are available or in development, clinical pathologists focus on how to choose the best method for routine diagnosis of lung cancer. There are many domestic and international authoritative guidelines recommend ALK detecting assays, technological process and relative standard. In the current review, we summarize the diagnostic tests available and the special sample types that could be used to identify patients with ALK-positive NSCLC.

## 1 间变性淋巴瘤激酶（anaplastic lymphoma kinase, ALK）与ALK阳性非小细胞肺癌（non-small cell lung cancer, NSCLC）

ALK是一种受体酪氨酸激酶，与白细胞酪氨酸激酶（leukocyte tyrosine kinase, LTK）属于同一亚家族，均为胰岛素受体（insulin receptor, IR）超家族成员。ALK的正常生理功能目前尚不明确，但研究^[1]^发现ALK主要表达于发育中的中枢和外周神经系统，说明ALK对神经系统的正常发育和功能具有作用。此外，ALK易位在肿瘤的发生、发展过程中起到了关键作用。1994年ALK首次以融合蛋白NPM（核磷蛋白）-ALK的形式在间变性大细胞淋巴瘤（anaplastic large cell lymphoma, ALCL）细胞系中被发现^[2]^，目前已有超过20种不同的ALK易位在多种癌症中已发现，包括ALCL（发生率60%-90%）、炎性肌纤维母细胞性肿瘤（inflammatory muscle fiber mother cell tumors, IMT）（发生率50%-60%）、NSCLC（发生率3%-7%）、结直肠癌（colorectal carcinoma, CRC）（发生率0%-2.4%）、乳腺癌（发生率0%-2.4%）和其他发生率很低的癌症^[3]^。

在部分NSCLC中，棘皮动物微管结合蛋白4（echinoderm microtubule-associated protein like 4, *EML4*）基因与*ALK*基因形成*EML4-ALK*融合基因，并在2007年由Soda教授^[4]^首次证实为NSCLC的驱动基因。随后，由于靶向抑制剂克唑替尼的出现，证实其对于存在*ALK*融合基因的NSCLC患者具有显著疗效而备受关注^[5]^。2009年-2011年，陆续研究包括2013年制定的《中国ALK阳性非小细胞肺癌诊断专家共识》逐步将*ALK*融合基因阳性的肺癌列为NSCLC的一个特定分子亚型，并将此类疾病统称为ALK阳性NSCLC^[5-7]^。ALK阳性NSCLC约占所有NSCLC的5%左右，在腺癌、年轻患者（< 60岁）以及不吸烟的人群中发生率较高，人种之间发生率无差异^[4, 6, 8-11]^。

## 2 ALK阳性NSCLC诊断适宜人群

有报道^[12]^显示ALK阳性NSCLC患者接受靶向治疗后，中位生存期可达到4.3年。由于ALK抑制剂治疗可大大延长晚期ALK阳性NSCLC患者的生存期，因此，如何最大程度筛选出ALK阳性患者成为临床靶向治疗的重要前提。国内外已有多篇指南或专家共识可用于指导ALK阳性NSCLC的诊断，其中较为重要的指南总结如表 1。对于ALK阳性NSCLC患者的适宜人群，总体上而言，各个指南均推荐腺癌或者含腺癌成分的NSCLC患者都需要检测ALK。对于不含腺癌成分的患者，各个指南则推荐不一。其问题主要集中于鳞癌患者是否需要检测。一般而言单纯的肺鳞癌不存在或存在很低的*ALK*基因融合几率，有研究^[13]^发现在1, 400例肺鳞癌患者中*ALK*融合基因的发生率约为1.3%，个别研究数据显示*ALK*基因融合几率还会偏高。这一差异主要是由于肺癌是一种高度异质性的肿瘤类型，形态不典型的鳞癌与腺癌的诊断本就存在一定的不确定性，即使结合免疫表型检测结果，病理同行间也会存在一定的判断与分析上的差异，所以造成鳞癌亚型诊断的偏差。同时，部分晚期患者仅可提供小活检标本用于诊断组织学分类，而仅仅依靠单点活检的小活检标本，对于一部分鳞癌或者腺癌类型的诊断更是十分困难。基于以上原因，部分指南推荐不吸烟（腺癌好发）、仅可提供小活检标本的鳞癌患者也应进行ALK检测。

**1:** 国内外ALK阳性NSCLC诊断指南或共识^[7, 9-12]^ Domestic and international diagnostic guidelines or consensus for ALK-postive NSCLC^[7, 9-12]^

Guidelines or consensus	ALK detecting group	Recommend detecting assay
The diagnosis and treatment guideline of chinese patients with egfr gene active mutation and alk fusion gene-positive non-small cell lung cancer (2014 version)^[9]^	All of NSCLCpatients with adenocarcinoma component	ALK break apart FISH probe method; RT-PCR or IHC by authority; the routine IHC should be used for screening
Chinese expertconsensus of ALK positive diagnosis in nonsmall cell lung cancer^[7]^	NSCLC patients with potential possibility of *ALK* fusion gene should be suggest to detect *ALK* rearrangement	Vysis ALK break apart FISH probe method; RT-PCR; the routine IHC should be used for screening
NCCN clinical practice guidelines in oncology for non-small cell lung cancer 2014 version 3^[10]^	NSCLC patients with non squamous cell carcinoma, and non smoking patients with Squamous cell carcinoma, or patients with small biopsy specimens or mixed pathologic types	FISH
IASLC atlas of ALK testing in lung cancer^[11]^	All of NSCLCpatients with adenocarcinoma component; patients with small biopsy specimens; non NSCLC patients with one of the following conditions: young, or mild/not smoking, *EGFR* mutation negative	Vysis ALK break apart FISH probe method; IHC method that compared with FISH with high consistency should be used for screening
CAP/IASLC/AMP EGFR and ALK detection guideline^[12]^	All of NSCLCpatients with adenocarcinoma component; NSCLC patients that can not be ruled out adenocarcinoma component	Vysis ALK break apart FISH probe method; IHC method that compared with FISH with high consistency should be used for screening
ALK: anaplastic lymphoma kinase; NSCLC: non-small cell lung cancer; EGFR: epidermal growth factor receptor; IHC: immunohistochemistry; FISH: fluorescence *in situ* hybridization; NCCN: National Comprehensive Cancer Network; RT-PCR: reverse transcription-polymerase chain reaction.		

另一方面，通常而言NSCLC的驱动基因是排他性存在的。在我国大陆和台湾地区表皮生长因子受体（epidermal growth factor receptor, *EGFR*）、*KRAS*均为野生型的腺癌中，ALK阳性比例高达30%-42%^[14, 15]^。因此，在国际肺癌研究协会（International Association for the Study of Lung Cancer, IASLC）的ATLAS指南中，对于已经明确*EGFR*基因突变阴性的患者，无论是否是鳞癌，均应检测*ALK*融合基因，以免患者失去治疗机会。

## 3 ALK阳性NSCLC的诊断方法

从机制而言，*EML4-ALK*基因融合通常为2号染色体短臂倒位突变[inv(2)(p21p23)]形成，并编码含EML4的氨基末端和ALK的胞内酪氨酸激酶生成融合蛋白。同时，ALK还可以与其他融合伴侣（如KIF5B、KLC1、PTPN3与STRN）形成融合基因并编码生成融合蛋白。因此多种不同的技术方法，包括最为常用的免疫组织化学（immunohistochemistry, IHC）、荧光原位杂交（fluorescence *in situ* hybridization, FISH）技术以及反转录聚合酶链反应法（reverse transcription-polymerase chain reaction, RT-PCR）等均可用于检测*ALK*融合基因。

### 3.1 分离探针荧光原位杂交（Break Apart FISH）

最初Soda与Rikova^[16]^分别通过PCR与蛋白组学的方法切入发现*EML4-ALK*融合基因存在于NSCLC中。但由于*ALK*融合基因的融合伴侣较多，且存在未知融合伴侣的可能性较大，因此采用分离探针的FISH法逐渐为大家所接受，并成为早期相关临床试验的伴随诊断方法。此外，目前所进行的临床研究也是以FISH法做为诊断的参照标准。

FISH分离探针试剂盒（Vysis ALK Break Apart FISH Probe Kit）是目前最经典的ALK阳性NSCLC的诊断试剂盒。该试剂盒设计了红与绿两个探针，分别标记为3’端的橘红色（部分文献也报道为红色，主要由于滤光镜不同），大约300 kb大小位于*ALK*基因的端粒端；5’端的绿色，大约442 kb大小，标记*ALK*基因的着丝粒端。因此，一旦*ALK*基因发生断裂重排或者倒转，红绿信号便出现分离。值得一提的是，分离探针的FISH方法无法判断ALK融合型。如图 1所示，对于ALK阳性的FISH判读流程，ATLAS指南中有比较详尽的描述。在无*ALK*融合基因表达的肿瘤细胞中，橘红色和绿色重叠为黄色或者相互粘合（两个信号之间的间隔小于两个信号的直径）；存在*ALK*融合基因表达的肿瘤细胞中，橘红色和绿色信号相互分离（间隔≥2个信号直径）。有经验的病理科医师挑选单个视野中的50个肿瘤细胞，如果 > 50%细胞出现分离信号则判读为阳性，如 < 10%细胞出现分离信号则判读为阴性，如10%-50%细胞出现分离信号，则需再次挑选50个肿瘤细胞，两者合并（100个肿瘤细胞）中 > 15%出现分离信号则为阳性，反之则为阴性。从上述流程可以看出，分离探针FISH法的诊断不但必须由经验丰富的病理科医师来完成，而且15%的Cut off值仅针对雅培的分离探针FISH试剂盒，对于其他种类的FISH方法，目前尚无定论。

**1:**
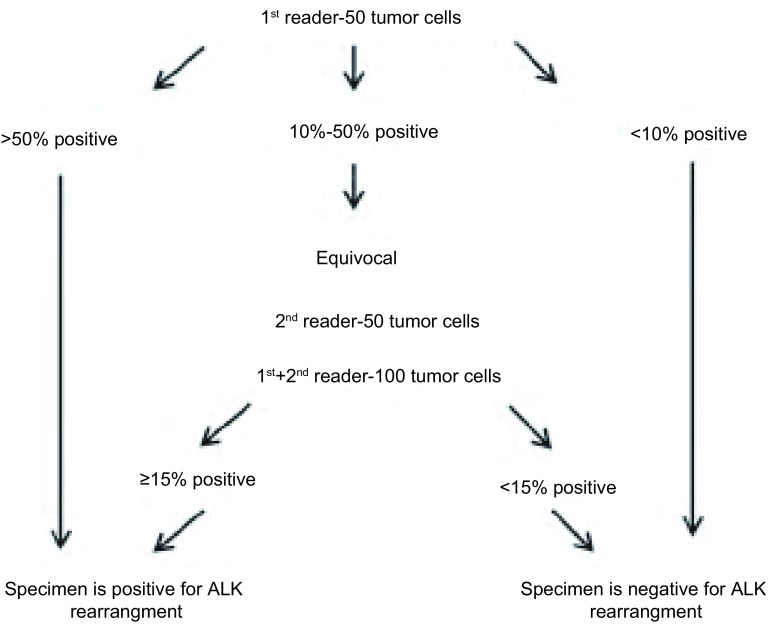
ATLAS ALK检测指南推荐的FISH诊断流程 Recommended scoring algorithm for ALK FISH of ATLAS

虽然FISH方法已作为ALK阳性NSCLC诊断的标准参照方法，但对于大规模检测ALK的临床需求而言，此方法也存在诸多不足。首先，对于15%这个Cut off值的设定一直存在争议^[17]^。有报道显示，8%的NSCLC患者其分离信号表达的肿瘤细胞数在10%-15%之间，加之肿瘤存在异质性，因此这部分患者单纯依靠FISH无法确切排除为非ALK阳性患者；由于方法本身的缘故，FISH检测还存在假阴性；另外，FISH检测的成本昂贵且操作较为复杂，对于检测对象而言其增加的医疗成本也可见一斑。综上所述，FISH法较适合于作为参照的标准方法，而应用于临床ALK阳性NSCLC的常规筛查则有较多限制。

### 3.2 IHC

基于FISH存在的一些问题，较多学者探索了IHC检测ALK蛋白的可行性，取得了可喜的结果。最早在淋巴瘤中用于检测ALK蛋白的ALK1抗体已经被证实在NSCLC中无法适用^[18]^。而之后研究^[19-21]^发现的D5F3、5A4等抗体在一定程度上具有较高的敏感性和特异性。在中国临床肿瘤学会（Chinese Society of Clinical Oncology, CSCO）专家共识中^[7]^，专家组总结多项研究，结果显示常规IHC 3+、IHC 2+、IHC 1+的患者与FISH的一致率达到97.4%、62.5%、14.3%，IHC假阴性率为0。同样，在国外的另一项大样本临床研究ETOP项目^[22]^中，ALK IHC 3+、IHC 2+、IHC 1+与FISH的一致率分别为90.9%、60.0%、4.2%，且IHC假阴性率同样为0。研究表明，297例肺腺癌患者的样本使用CTS’s D5F3抗体免疫组化方法检测和FISH法检测*ALK*重排比对，发现使用CTS’s D5F3抗体免疫组化方法可以达到100%的敏感性和98%的特异性。另外IHC方法由于其操作简单、判读相对简单、费用较低，所以IHC作为大范围常规筛查的手段，已在国内外达成共识。

为提高IHC用于诊断ALK阳性NSCLC的特异性，2013年罗氏/Ventana公司在BenchMark平台上进一步优化了D5F3抗体检测试剂盒及其检测流程。其优势在于一抗孵育后的充分水洗过程，最大程度的解决了特异性的问题。与此同时，该试剂盒加入两级放大的Optiview系统，使得原本表达非常弱的ALK融合蛋白得以呈现较为明显的染色效果，并以NSCLC细胞株来源的二合一质控片作为内部对照，最大程度地保证了结果的可靠性。该方法阳性结果判读标准为肿瘤细胞胞浆中出现簇状综黄色强染色颗粒，胞膜着色视为阴性结果。大部分阳性肿瘤显示均匀强表达如图 2（图 2A、图 2B），个别阳性病例则由于肿瘤异质性表现为表达强度的不均一性，如图 2（图 2C、图 2D）（图片来自罗氏公司提供的结果判读说明）。多项Ventana IHC与FISH检测结果对照研究表明二者的一致率为94%-100%，如表 2所示。基于此数据，欧盟以及中国已经批准Ventana IHC用于检测*ALK*重排，并可指导克唑替尼治疗。

**2:**
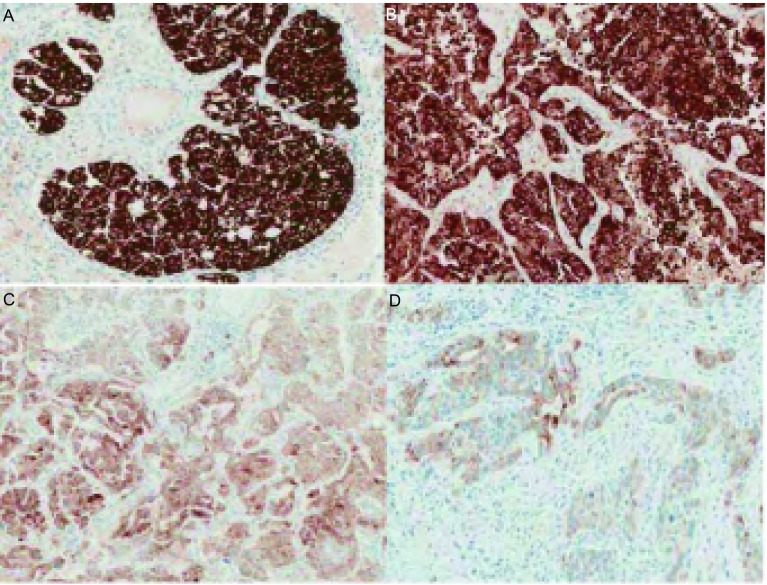
Ventana IHC抗ALK（D5F3）兔单克隆抗体检测肺腺癌ALK染色阳性病例（×200）。A、B：大部分阳性肿瘤显示均匀强表达；C、D：个别阳性病例则由于肿瘤异质性表现为表达强度的不均一性。 Staining positive cases of Ventana ALK IHC with ALK rabbit monoclonal antibody (D5F3) in lung adenocarcinoma (×200). A, B: Most positive tumors showed strong and regular expression; C, D: Due to tumor heterogeneity, individual positive cases showed expression of heterogeneity.

**2:** Ventana IHC与FISH对照研究结果一览 Comparative study result of Ventana IHC and FISH

Authors		FISH		Total	Sensitivity	Specificity	Concordance rate
		Positive	Negative				
Wang J, *et al*^[23]^	Positive	46	7	53	100%	98%	98%
	Negative	0	377	377			
Minca EC, *et al*^[24]^	Positive	32	0	32	100%	100%	100%
	Negative	0	217	217			
Wynes MW, *et al*^[25]^	Positive	40	2	42	90%	96%	94%
	Negative	4	53	57			
Ali G, *et al*^[26]^	Positive	18	0	18	90%	100%	99%
	Negative	2	503	505			

### 3.3 RT-PCR

基于PCR平台检测ALK基因重排技术近年来发展迅速。2008年日本的Takeuchi等^[27]^通过单管多重逆转录聚合酶链技术和测序检测了手术切除的253例肺腺癌样本，与金标准FISH法比对，发现有11例样本有*ALK*基因重排，阳性率为4.35%。2010年国内吴一龙教授课题组^[28]^的研究采用应用RACE-coupled PCR测序的方法检测分析*ALK*的融合变异。该方法采用合有逆转录的RACE结合两轮PCR技术来富集扩增ALK的融合变异体。该方法敏感性高，能进一步通过测序的手段明确*ALK-EML4*融合基因的类型，并能检测到其他任何基因与ALK的融合类型，但由于该方法实验步骤繁琐，并不适用于临床推广。最近德国的Robesova等^[29]^通过特异的qPCR法和分离探针FISH的方法对比检测46例NSCLC样本，发现用特异qPCR法检测出17/46（37.0%）例ALK阳性样本，而FISH法仅检测出8/46（17.4%）例ALK阳性样本，故特异qPCR法展现出快速、高敏感性、易于商业推广的优势。目前qPCR方法在*EGFR*突变检测中，已经成为标准方法之一。但对于检测*ALK*重排来说，RT-PCR法更加简便可行，国内外亦有诸多研究证实了RT-PCR法的可靠性。除此以外，RT-PCR方法的优势还在于与FISH和IHC方法相比，RT-PCR方法可确定ALK的融合型，虽然临床意义不大，但是随着研究的深入，ALK融合型与疗效之间的关系也值得我们进一步去探索。在这一点上，EGFR基因19外显子与21外显子突变患者接受EGFR抑制剂治疗获得的疗效不同已为我们提供一定的启示^[30]^。其次，对于新鲜肿瘤组织而言，由于RNA质量可以得到保证，RT-PCR方法检测*ALK*重排应该是首选。

但RT-PCR法存在一定的局限性。首先由于RT-PCR需提供高质量的RNA样本来确保检测结果准确，而临床上大部分的检测样本为石蜡组织，RNA降解严重，影响逆转录的效率，从而可能导致ALK的假阴性结果。但随着商业化提取和检测试剂盒的日益改进，上述问题也在逐渐被克服。另外，由于PCR引物设计只能针对已知的融合基因型，RT-PCR方法通常无法囊括所有的融合型。因此，国内外指南对于此方法的推荐程度不一。国内的中国EGFR与ALK阳性NSCLC诊断及治疗指南（2014版）与CSCO中国专家共识均推荐使用获得认证的RT-PCR试剂盒用于临床检测*ALK*融合基因。而国外的ATLAS指南与CAP/IASLC/AMP9指南则不推荐RT-PCR方法。

### 3.4 其他方法

#### 3.4.1 显色原位杂交（chromogenic *in situ* hybridization, CISH）

有少量研究用CISH的方法检测*ALK*基因重排。最近一项研究表明，以雅培分离探针为金标准，比对CISH的方法，发现CISH方法有5%的假阴性，即19例FISH阳性的样本使用CISH的方法检测，有1例未能检测出来。CISH法检测ALK的优势是实验过程较简单，可以同时兼顾观察ALK阳性信号和肿瘤的组织形态，但局限是CISH对于5’和3’端的蛋白剪切体的检测灵敏度下降，存在假阴性结果。目前该方法还存在一定局限性。

#### 3.4.2 二代测序（next generation sequencing, NGS）

NGS的平台有PacBio RS（Pacific Biosciences, Menlo Park）、Ion Torrent PGM（Life Technologies）和Illumina HiSeq（Illumina, San Diego）等。目前已证实使用二代测序技术用来检测基因扩增和基因重排是可行的，已有研究通过NGS技术发现NSCLC中几种新的*ALK*基因重排和肺癌中其他新的融合基因。

然而，NGS的临床应用仍存在较大争议。和基于PCR平台的检测方法一样，目前NGS的临床应用实践中也必须要注意怎样避免漏检掉有意义的未知的*ALK*基因重排的类型。使用NGS检测*ALK*基因重排的优势是，对于病理医生来说在考虑给患者开展基因检测时，通过一次多通道实验就能获得多种已知的基因突变、基因重排和基因扩增的信息。传统的检测平台，多项基因检测将耗费大量的患者样本，而NGS检测可以仅使用少量的样本。所以NGS技术的多通量、便捷性和节约检测样本的效益等优势使这项技术有巨大的应用潜力。到目前为止，并没有更多的实验数据比对*ALK*基因重排的金标准FISH检测和二代测序的差异，而且NGS技术的高昂费用、判读的标准及后续验证等众多问题仍待解决，期待不远的将来这项技术可以更广泛的应用于临床靶基因的检测。

## 4 适用于检测ALK阳性NSCLC的标本选择

通常而言，使用于ALK检测的标本为常规10%中性福尔马林固定石蜡包埋（formalin-fixed and parrffin-embedded, FFPE）肺癌标本，由于存在蛋白或RNA降解可能，FFPE制备后切片储存大于3个月的不建议Ventana IHC检测，固定标本大于2年的不建议RT-PCR检测。对于新鲜肿瘤组织样本，RT-PCR通常为首选的诊断方法。其他少见检测样本有细胞学和血液样本，有些还在临床研究阶段。

### 4.1 细胞学样本

就晚期患者而言，40%的患者无法提供活检组织样本，因此，细胞学标本诊断ALK重排的方法学是目前值得探讨的热点问题。细胞学的样本来源通常为体表淋巴结穿刺、CT引导下细针穿刺、恶性积液、超声引导下支气管镜细针穿刺、各种分泌物或者灌洗液等。目前通行的方法是将细胞学样本制成包埋的细胞蜡块，因为这样做既有利于长期保存，也可按照组织蜡块的步骤进行相关检测，包括使用Ventana IHC和FISH方法。但对于细胞学样本中肿瘤细胞数量较少时，或存在瘤细胞与非肿瘤反应性细胞如间皮细胞等难以鉴别时，对FISH结果的判读则提出较高要求。因此对于细胞学样本，IHC方法与RT-PCR方法可能更为适合，也有使用5A4抗体对细胞学蜡块进行ALK IHC检测的研究^[19, 31, 32]^报道，总体而言，敏感性与特异性分别达到了93.3%与96.0%，且检测成功率接近100%。亦有研究^[33]^报道利用RT-PCR对细胞学样本进行ALK检测，在36例患者中的检测结果与组织样本相比一致率达到了100%。由于技术平台不同，RT-PCR检测对于细胞学样本特别是离心浓缩后的恶性胸水样本具有一定优势。

### 4.2 血液样本

血液样本中检测*EGFR*突变已为大部分临床和病理科医师接受，欧盟也已批准采用血液循环肿瘤细胞DNA（ctDNA）检测方法评估*EGFR*突变状态以用来监测指导吉非替尼的治疗。但是，基于PCR平台的检测方法对于血液检测ALK仍存在一定的先天不足。主要原因是目前RT-PCR均是在RNA层面检测*ALK*融合基因，而在血液中存在大量RNA酶，大部分肿瘤RNA释放入血后都已降解。因此，最近*Nat Med*杂志报道了美国学者^[34]^使用二代测序方法检测血液中游离DNA的*ALK*融合基因表达，结果报道4例阳性患者，其中2例患者接受克唑替尼治疗均获得良好疗效，从而提示此方法可能具有一定的应用前景。

从循环肿瘤细胞的层面使用FISH方法检测ALK，也得到一些可喜的结果。来自法国的Pailler^[35]^使用FA-FISH（filter-adapted FISH）方法检测18例ALK阳性和14例ALK阴性NSCLC患者的循环肿瘤细胞。结果发现所有ALK阳性患者中，每1 mL血液可检测到 > 4个循环肿瘤细胞存在ALK分离信号表达，并且这些存在*ALK*融合基因的循环肿瘤细胞均存在间质转化亚型，提示这部分细胞具有高度侵袭性。

## 5 总结

ALK阳性NSCLC是NSCLC的一类分子亚型，最大化的发挥ALK抑制剂如克唑替尼的疗效有赖于准确、客观的检出ALK阳性患者，尽管目前在多数检测指南中FISH仍然作为检测的金标准，但临床实践中对常用的三种检测方法FISH、Ventana IHC及RT-PCR进行比较发现三种方法特异性均较好，但FISH的敏感性最低，而且已经有数据显示FISH检测ALK阴性而Ventana IHC阳性的患者可以从克唑替尼治疗中获益（资料待发表）。根据中国专家制定的指南和共识，分离探针FISH、Ventana IHC、已经国家认证的RT-PCR均可用于ALK阳性NSCLC诊断。对于没有Ventana IHC检测平台的实验室而言，使用D5F3进行IHC仍然可以作为一个有效的*ALK*重排检测的初筛方法，阳性或可疑阳性病例需FISH或RT-PCR法作为有效的补充验证方法。

## References

[1] Pulford K, Lamant L, Espinos E (2004). The emerging normal and disease-related roles of anaplastic lymphoma kinase. Cell Mol Life Sci.

[2] Morris SW, Kirstein MN, Valentine MB (1994). Fusion of a kinase gene, *ALK*, to a nucleolar protein gene, *NPM*, in non-Hodgkin's lymphoma. Science.

[3] Grande E, Bolós MV, Arriola E (2011). Targeting oncogenic *ALK*: a promising strategy for cancer treatment. Mol Cancer Ther.

[4] Soda M, Choi YL, Enomoto M (2007). Identification of the transforming *EML4-ALK* fusion gene in non-small-cell lung cancer. Nature.

[5] Kwak EL, Bang YJ, Camidge DR (2010). Anaplastic lymphoma kinase inhibition in non-small-cell lung cancer. N Engl J Med.

[b6] 6Shaw AT, Kim DW, Nakagawa K, *et al*. Phase 3 randomized study of crizotinib versus pemetrexed or docetaxel chemotherapy in advanced, ALK-positive NSCLC (PROFILE 1007). Vienna, Austria: European Society for Medical Oncology. 2012 ESMO Abstract LBA1.

[7] Zhang XC, Lu X, Zhang L (2013). Chinese expert consensus of anaplastic lymphoma kinase (ALK) positive diagnosis in non small cell lung cancer. Zhonghua Bing Li Xue Za Zhi.

[8] Wong DW, Leung EL, So KK (2009). The *EML4-ALK* fusion gene is involved in various histologic types of lung cancers from nonsmokers with wild-type *EGFR* and *KRAS*. Cancer.

[9] Chinese Association of Oncologists, Chinese Society for Clinical Cancer Chemotherapy (2014). The Diagnosis and Treatment Guideline of Chinese Patients with *EGFR* gene active mutation and *ALK* fusion gene-positive non-small cell lung cancer (2014 version). Zhonghua Zhong Liu Za Zhi.

[b10] 10National Comprehensive Cancer Network. NCCN clinical practice guidelines in oncology for non-small cell lung cancer version 3, 2014.

[11] Leighl NB, Rekhtman N, Biermann WA (2014). Molecular testing for selection of patients with lung cancer for epidermal growth factor receptor and anaplastic lymphoma kinase tyrosine kinase inhibitors: American society of clinical oncology endorsement of the college of American pathologists/international association for the study of lung cancer/association for molecular pathology guideline. J Clin Oncol.

[12] Crino L, Kim D, Riely GJ (2011). Initial phase Ⅱ results with crizotinib in advanced ALK-positive non-small cell lung cancer (NSCLC): PROFILE 1005. J Clin Oncol.

[b13] 13Tsao MS, Hirsch FR, Yatabe Y. IASLC atlas of ALK testing in lung cancer. International Association for the Study of Lung Cancer, 2013.

[14] Zhang X, Zhang S, Yang X (2010). Fusion of *EML4* and *ALK* is associated with development of lung adenocarcinomas lacking EGFR and KRAS mutations and is correlated with ALK expression. Mol Cancer.

[15] Wu SG, Kuo YW, Chang YL (2012). *EML4-ALK* translocation predicts better outcome in lung adenocarcinoma patients with wild-type EGFR. J Thorac Oncol.

[16] Rikova K, Guo A, Zeng Q (2007). Global survey of phosphotyrosine signaling identifies oncogenic kinases in lung cancer. Cell.

[17] Camidge DR, Skokan M, Kiatsimkul P (2013). Native and rearranged *ALK* copy number and rearranged cell count in non-small cell lung cancer: implications for ALK inhibitor therapy. Cancer.

[18] Mino-Kenudson M, Chirieac LR, Law K (2010). A novel, highly sensitive antibody allows for the routine detection of *ALK*-rearranged lung adenocarcinomas by standard immunohistochemistry. Clin Cancer Res.

[19] Martinez P, Hernández-Losa J, Montero MÁ (2013). Fluorescence in situ hybridization and immunohistochemistry as diagnostic methods for ALK positive non-small cell lung cancer patients. PLoS One.

[20] Paik JH, Choi CM, Kim H (2012). Clinicopathologic implication of ALK rearrangement in surgically resected lung cancer: a proposal of diagnostic algorithm for *ALK*-rearranged adenocarcinoma. Lung Cancer.

[21] McLeer-Florin A, Moro-Sibilot D, Melis A (2012). Dual IHC and FISH testing for *ALK* gene rearrangement in lung adenocarcinomas in a routine practice: a French study. J Thorac Oncol.

[22] Shan L, Lian F, Guo L (2014). Combination of conventional immunohistochemistry and qRT-PCR to detect *ALK* rearrangement. Diagn Pathol.

[23] Wang J, Cai Y, Dong Y (2014). Clinical characteristics and outcomes of patients with primary lung adenocarcinoma harboring *ALK* rearrangements detected by FISH, IHC, and RT-PCR. PLoS One.

[24] Minca EC, Portier BP, Wang Z (2013). ALK status testing in non-small cell lung carcinoma: correlation between ultrasensitive IHC and FISH. J Mol Diagn.

[25] Wynes MW, Sholl LM, Dietel M (2014). An international interpretation study using the ALK IHC antibody D5F3 and a sensitive detection kit demonstrates high concordance between ALK IHC and ALK FISH and between evaluators. J Thorac Oncol.

[26] Alì G, Proietti A, Pelliccioni S (2014). ALK rearrangement in a large series of consecutive non-small cell lung cancers: comparison between a new immunohistochemical approach and fluorescence *in situ* hybridization for the screening of patients eligible for crizotinib treatment. Arch Pathol Lab Med.

[27] Takeuchi K, Choi YL, Soda M (2008). Multiplex reverse transcription-PCR screening for *EML4-ALK* fusion transcripts. Clin Cancer Res.

[28] Zhang X, Zhang S, Yang X (2010). Fusion of *EML4* and *ALK* is associated with development of lung adenocarcinomas lacking EGFR and KRAS mutations and is correlated with ALK expression. Mol Cancer.

[29] Robesova B, Bajerova M, Liskova K (2014). TaqMan based real time PCR assay targeting *EML4-ALK* fusion transcripts in NSCLC. Lung Cancer.

[b30] 30Yang JCH, Sequist LV, Schuler MH, et al. Overall survival (OS) in patients (pts) with advanced non-small cell lung cancer (NSCLC) harboring common (Del19/L858R) epidermal growth factor receptor mutations (*EGFR* mut): pooled analysis of two large open-label phase Ⅲ studies (LUX-Lung 3[LL3] and LUX-Lung 6[LL6]) comparing afatinib with chemotherapy (CT). J Clin Oncol, 2014, 32(5 suppl): abstr 8002.

[31] Savic S, Bode B, Diebold J (2013). Detection of ALK-positive non-small-cell lung cancers on cytological specimens: high accuracy of immunocytochemistry with the 5A4 clone. J Thorac Oncol.

[32] Moreira AL, Hasanovic A (2012). Molecular characterization byimmunocytochemistry of lung adenocarcinoma on cytology specimens. Acta Cytol.

[33] Mitiushkina NV, Iyevleva AG, Poltoratskiy AN (2013). Detection of EGFR mutations and *EML4-ALK* rearrangements in lung adenocarcinomas using archived cytological slides. Cancer Cytopathol.

[34] Newman AM, Bratman SV, To J (2014). An ultrasensitive method for quantitating circulating tumor DNA with broad patient coverage. Nat Med.

[35] Pailler E, Adam J, Barthélémy A (2013). Detection of circulating tumor cells harboring a unique *ALK* rearrangement in ALK-positive non-small-cell lung cancer. J Clin Oncol.

